# A note on motor skill acquisition in mild and moderate Down syndrome individuals

**DOI:** 10.1186/s41155-017-0060-1

**Published:** 2017-03-15

**Authors:** Roberto Gimenez, Marcelo Luis Marquezi, Ernani Xavier Filho, Edison de J. Manoel

**Affiliations:** 1 UNICID - Grupo de Estudos sobre o Comportamento Motor e Intervenção Motora, São Paulo, SP - Rua Cesário Galeno, 448, Tatuapé, São Paulo 03071-000 Brazil; 2 UEL - Londrina, PR - Rodovia Celso Garcia - Km 380, s/n, Londrina, Paraná 86057-970 Brazil; 3 USP - Grupo de Estudo do Desenvolvimento da Ação e Intervenção Motora, São Paulo, SP - Avenida Professor Mello Moraes, 65 - Cidade Universitária, Butantã, São Paulo 05508-030 Brazil

**Keywords:** Down syndrome, Target directed movement, Movement timing, One-target advantage

## Abstract

This study investigated the acquisition of a serial motor skill in individuals with Down syndrome with two levels of handicap, mild group (mean age = 14.5 years, SD = 2.3, 7 individuals) and moderate group (mean age = 15.2 years, SD = 3.2, 7 individuals). The task involved single-arm sequential movements to five. The measures to access performance were overall sequence error, reaction time, and total movement time. To evaluate action program, formation variability of sequencing and relative timing variability were considered. Although there was no clear practice effect, the results showed that the level of handicap led to different strategies to plan and control the actions. The moderate group presented a less stable action program expressed in the variability in sequencing and timing. Their longer reaction times also suggest a heavy demand on central processing in accord with the one-target advantage hypothesis and also due to memory deficits to select and plan movements.

## Background

One common assumption in regard to the motor behavior of handicapped people is that they are different from the so called normal individuals, difference that is invariably taken as synonymous of the handicap individual being slower, more variable, inaccurate, and inefficient than their normal counterpart. The rationale underlying this judgement stems out of a common way of doing scientific psychology known as the nomothetic method (cf. Valsiner 1986). This method assumes that behavioral phenomena showed marked regularities that can be interpreted as a given normal pattern. Hence, there is an ideal child, an ideal developmental stage or pattern, and everything else outside the range of this ideal pattern is considered abnormal and defective. If this way of thinking gave good results in revealing typically developing patterns, it overlooked, among other things, how handicapped individuals develop or simply neglected their development, as they were treated as having pathology. From an epistemological point of view, the use of the nomothetic method in medicine was criticized and its limits have been pointed out by a long time ago by Canguilhem (2012). This method also reveals what Gould (1996) remarks as our orthodox and fallacious way of treating “particular or abstractions quite often biased” (p. 15) as hallmarks of a developmental trend while all observed variation is simply treated as noise or irrelevant to the understanding of biological phenomena. Gould (1996) makes the case that we should be looking for variation and its changing pattern of spread through time. Therefore, it is quite limited to study the behavior of handicapped people in regard to a normal behavior. Actually, in the long run, this is quite inappropriate, as what one should look for is the variability presented among individuals which means asking how the biological organization is alike among the different.

Some studies have argued that motor control systems have to make choices to deal with everyday motor problems and they do soon the bases of coordinative rules (Latash and Anson 1996; Latash 2007). The task of motor control researchers is to unveil them. One interesting hypothesis that derives from Latash and Anson (1996) proposition is that the coordinative rules for atypical populations are simply different from the typical (normal) population; their central nervous system’s priorities are different and the resulting motor patterns are not pathological but adaptive. From this perspective, we know very little about motor behavior of the handicap and the ways this population organize them. Bearing this in mind, the present paper addresses the question of how Down syndrome individuals acquire a serial motor skill. The interest is not to compare them with typical, normal population, but to invest in gathering information on how these individuals with different degrees of their condition (mild, moderate, and heavy) set out to solve motor problems, hence unveiling to some extent the coordinative rule they abide for.

Latash and Anson (1996) have pointed out that the research strategy on motor difficulties leading to the so called abnormal behavior should unravel the adaptive strategies behind what was believed to be limitations. In other words, the goal should be to look for what handicapped people are able to do rather than on their limitations, some of which are inevitable as they are due to neural and motor impairments. When one adopts this strategy comparisons between normal people and Down syndrome people are of less importance than looking for differences within the Down syndrome samples considering their level: mild, moderate, and heavy.

Literature suggests that motor control strategies developed by handicapped individuals show large behavior dynamism, as well as the wealth of resources that guarantee their adaptation to the environment. Thus, the insistence to compare their behavior with typical individuals on some occasions may not be sufficiently enlightening. The criteria underlying the nomothetic designs that are related to measurement and which are based on age and gender parameters for the separation of groups should be revisited in the study of handicapped people (Bouffard 1993; Bouffard et al. 1998).

Gimenez and Manoel (2005) suggest that one of alternatives to deal with this demand would give more attention to the strategies of motor control in these individuals, ensuring their characterization by analysis of effective ways of adaptation to the contexts and demands of the tasks, without necessarily adopt as reference the behavior of populations considered typical.

It has been suggested that Down syndrome (DS) individuals while performing rapid discrete and serial actions adopt an adaptive control strategy relying more on feedback control or online control and less on feedforward control/or pre-programming (Almeida et al. 2000). For instance, Charlton et al. (1996) have studied a sample of seven DS children (average age of 9) performing a reach and grasp task. They focused on the kinematic pattern of the arm, forearm, and wrist. From the velocity profiles, it was possible to calculate the number of movement unities. Following the work of Claes von Hofsten (cf. Von Hofsten 1991) in reaching and grasping tasks, it is accepted that the higher the number of movement units more likely a feedback programming strategy is in use, while the reverse indicates a reliance on feedforward programming. Charlton et al. (1996) found that DS children showed greater number of movement units in comparison to matched control groups (composed by typically developing children). Recently, Vimercati et al. (2013) expanded Charlton et al.’s results by looking at 22 DS adult subjects performing a serial task (tapping). The task did not have the grasping component giving better condition to test the hypothesis of an adaptive control strategy since Charlton et al. speculated that their results were due to the demands of the grasping component that warrants greater accuracy to complete the action. Vimercati et al. (2013) made a thorough motion analysis that allowed them to have elements to describe the movement strategies used; hence, they tracked the trajectories of the elbow, wrist, and finger, also the rotation of the trunk. In comparison with matched normal adult subjects, DS individuals showed greater range of motion at the trunk while keeping the elbow stiff. The adaptive control strategy used by DS individuals was also highlighted by the greater number of movement unities given by the velocity profile of the wrist which means greater reliance on feedback control.

Lawrence et al. (2013) conducted a study bringing evidence that central factors rather peripheral ones play a major role in the deficits observed in the motor performance of individuals with Down syndrome. They specifically tested the one-target advantage hypothesis (OTA) in sequential aim movements. This hypothesis states that movement time to an initial target is longer when there is a subsequent movement to a second target. This will impact also the reaction time to start the sequence. It will be longer when there is more than one target. Lawrence et al. (2013) asked DS individuals to perform a single target movement, a two-target movement performed by a single arm, and a two-target movement performed bi-manually (the first movement was performed with one arm, and the second movement with the other arm). OTA was observed for DS individuals in a similar strategy observed with the other participants in the study (typically developing individuals and individuals with an undifferentiated intellectual disability). The comparison between the single-arm and two-arm two-target response indicated no difference in the OTA effect for DS individual giving evidence that longer reaction and movement times to start the sequential aiming movements are due to central processes.

Taking together, the results suggest that motor planning and programming are somehow restricted in DS individuals; hence, their resulting movement patterns are controlled mostly online. Rather than being a defect, this is an adaptive strategy to deal with some limitations to plan ahead and also to pre-program motor units into one-single and detailed motor program. These limitations have been pointed out before by a number of authors (e.g., Wade et al. 1978; Seyfort and Spreen 1979; Anwar 1981; Kerr and Blais 1985; 1987; Dummer 1985; Inui et al. 1995; Jarrold et al. 2009). DS individuals would show some diverse structural organization in regions involved in motor planning (the pre-frontal lobe) and programming (the cerebellum and basal ganglia) (cf. Jeannerod 1997; Stoodley and Schmahmann 2009; Milojevich and Lukowski 2016). These differences would be identified especially in sequential tasks and in dual task conditions (Lanfranchi et al. 2012; Lanfranchi et al. 2015).

In this sense, it could be hypothesized that DS individuals during practice will not show evidence for motor program formation and programming will play a secondary role in the performance as in fact has been shown by the Charlton et al. and Vimercati et al. studies. Nevertheless, these two studies focused on motor control, i.e., the participants did not have practice in the task apart from what was necessary to become familiar with the experimental situation. Gimenez et al. (2006) looked at action program formation in four mild DS individuals (mean age = 19.5 months) who practiced a graphic task. DS individuals did benefit from practicing a graphic task taken by the decrease on total movement time to perform the task showing very similar performance with that of the typically developing children and adults.

However, there was no clear indication that an action program was formed because of the great variability observed in the relative timing of the strokes and in the sequencing of the strokes. Meanwhile, the typically developing individuals showed stability particularly in the sequencing of the action as a result of practice. One could argue that the lack of program formation during practice might be more evident, the greater the degree of mental handicap.

Admittedly, the literature presents some controversy about the nature of these difficulties of individuals with DS. Lanfranchi et al. (2010) argue about problems in the spatial aspects of motor tasks, identified in the sequencing of the different elements. On the other hand, more recently, Milojevich and Lukowiski (2016) postulate that these demands would be identified in tasks requiring temporal adjustments.

Carmelli et al. (2008) have also suggested that the degree of mental handicap needs to be investigated within a sample of DS individuals. This is considered to be a strategy to unveil the spread of variance among a population that quite often is treated as one sole group.

Apart from that, we need to investigate whether DS individuals will benefit from practice by elaborating and improving online control strategies or will then show more pre-programming in their actions. And also, whether this will be affected in any degree by the level of handicap, for instance in the comparison between the moderate and mild levels.

These concerns are raised by some studies (Schurink et al. 2012; Chen et al. 2014). Chen et al. (2014) adopted designs that relate levels of cognitive development and motor performance in DS individuals and concluded a strong correlation between cognition and motor performance. However, these same authors argue about the demand for studies that seek to investigate this relationship, especially in tasks involving motor planning.

The aim of the present study was to investigate how and whether DS individuals with two levels of handicap perform and learn a serial motor skill. The research questions were as follows: (1) Will DS individuals learn a sequential tapping task? (2) Will they show the formation of action program to perform a sequential tapping task? (3) Will the formation of an action program be constrained by the level of mental handicap (mild and moderate DS)?

## Method

### Participants

Fourteen DS individuals took part in the study; their chronological age range was 14.5 years for mild group (sd = 2.3) and 15.2 years for moderate group (sd = 3.2). They came from the same institution that cares for DS children and adolescent offering activities and training programs which means that all individual had similar levels of experiences and stimulation in a daily basis. They were equally distributed into two groups according to their degree of mental handicap: the mild group (*n* = 7) and moderate group (*n* = 7).

The criteria used to define the groups were based on the notes of the institution evaluated which takes into account the criteria of the American Association on Mental Retardation protocols (AAIDD 2010) and proposed by Richards et al. (2015).

All participants were voluntary following the informed consent form signed by their parents who were informed about the goals of the study. The research project was previously submitted and approved by the Committee of Ethics on Research from the School of Physical Education and Sport, University of São Paulo City, process no. 2009/57.

### Apparatus and experimental task

**Fig. 1:**
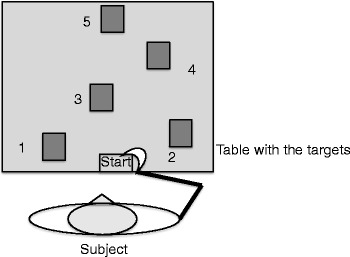
Experimental setting

The apparatus consisted of a table on top of which there were six touch sensitive plates spatially distributed as is represented on Fig. 1. The plates were connected to a notebook DELL Latitude D520 that registered the time and the order in which each plate was touched: (a) the total time to complete the task; (b) the order in which each plate was touched; (c) the movement time for touching the first and then the second plate. The software also controlled the beginning (by giving alert and then the start signal) and ending of a trial (once the subject has touched five plates, it registered the time taken and end the trial).

In the experimental task, the participant was standing in front of the table that was kept at a regular height according to the individual’s hip with his or her dominant hand resting on the starting plate. Once the computer gave a sound signal for starting, the individual had to touch as fast and as accurate as possible each plate in the order indicated by a number next to it. The individual was instructed to make full contact with the plate with distal portion of the hand. The order for touching defined the task sequence and the experimenter at the beginning of every trial presented it to the participant. The sequence was 1-3-5-2-4-5 in order to create a three part movement: part I [1-3-5], with the hand tapping in one direction in abduction; part II [5-2] that involved a reversal in the tapping with an abduction; part III [2-4-5] with tapping in one direction in abduction but in a different angle from part I. This spatial configuration and the sequence were chosen to create different components for the program. According to Young and Schmidt (1990), displacement variations in direction with reversals in discrete and serial movements implicate in the production of different programs or in the case of the present task, different sub-programs. One common strategy shown by typically developing children and young adults to deal with the information processing and control demands presented in this task is to pre-program the actions as far in advance as possible Fischman (1984).

### Procedure

The experiment session was conducted individually in a quiet room specially prepared for data collection. First, the experimenter said to the participant that he would be playing a game of tapping as fast as possible with a couple of plates on the table. Second, the experimenter performed a task sequence (the same of the experiment) and asked the participant to do the same. This was repeated in case the participant gave any sign of misunderstanding. Third, the experimenter asked whether the participant had any doubt or query about the practice. Once it was clear that the participant understood what the task entailed, the experimental session was initiated. Before the attention signal, the experimenter showed him the task sequence, a procedure repeated before the beginning of every trial. If the participant started before the signal, a warning signal was given and the trial aborted. Spatial errors were not pointed out by the experimenter during a trial only after, unless the participant failed to touch a plate. In this case, the experimenter would say: you forgot to touch one (or more if it was the case) plate. This was necessary because the computer’s clock was programmed to stop only when the programmed number of plates was touched. After each trial, the experimenter gave information on the total time response pointing out whether there was improvement or not. He also gave information about errors in the sequence.

The practice consisted of 15 trials performed in one session. The interval between each trial was around 45 s to 1 min depending on the number of information that had to be given to the participant in regard to spatial errors that occurred.

### Measures and statistical analysis

The measures were thought to indicate two different aspects of the participants’ experience. One aspect was regarding their overall performance that gave indications on how they benefit from practice. Another aspect referred to data that allow some inference on whether they developed a strategy for pre-programming during their practice.

The overall performance was given by

1. *Overall sequence error* was the number trials in a block (five trials) in which the sequence order was wrong.
2. *Reaction time* is the time in milliseconds between the start signal and the moment that the hand left the start plate. Due to a problem in data collection for this measure, there are the results of the first 15 trials or three out of five blocks of practice.
3. *Total movement time* is the interval measured in milliseconds between the moment that the hand left the start plate and touched the last sensor target in the task sequence.

The programming strategy was given by

1. *Variability of the sequence* that was obtained by the number of times a sequence of touches was presented in a block of trials divided by five (total number of trials in a block). If the subject hits always the targets in the right sequence, he or she scored 0.2; otherwise, if the subject performed two different sequences in a block, he or she scored 0.4.
2. *Relative timing variability* was obtained by the mean of intra-subject standard deviation of relative timing of *t* aiming movements to the targets. Relative timing of a given aiming movement (between two targets) was expressed as proportion of the total movement time. Due to a problem in data collection for this measure, there are the results of the first 15 trials or three out of five blocks of practice.

In spite of being related to the second measure of the overall performance, the emphasis here is placed on the stability, or lack of it, that might be attained in the task sequence during practice. Sequencing is acknowledged as a classical feature of the representation of an action program since Lashley (1951), hence once it is stable, indicates that an outline of the task is being centrally represented (cf. Jeannerod 1997).

For the purpose of statistical analyses, the practice session was divided in five blocks with five trials. Experimental data are expressed as means ± SEM. Before statistical analysis, the variables were tested for normality by univariate (Cochran C, Hartley, Barlett) and Brown-Forsyhte tests. A two-way general linear model for repeated measures (groups × blocks) was used to identify differences between the experimental trials; when a significant F-ratio was obtained, the Tukey post hoc test with the Bonferroni correction was used to locate the differences. Comparison of variables between groups was conducted using one-way ANOVA and, as a post hoc test, the Tukey test. For all statistical analyses, significance was accepted at *p* < 0.05. Statistical analysis was performed using STATISTICA data analysis software system (version 8.0, StatSoft, Inc., 2007, USA).

## Results

### Overall sequence error

**Fig. 2:**
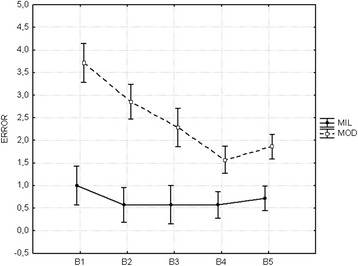
Mean overall sequence error

The overall sequence error showed changes during practice particularly for the moderate group, *F*_(1,12)_ = 19.030, *p* = 0.009 (Fig. 2). The mild group was more accurate than the moderate group. The Tukey post hoc test with the Bonferroni correction indicated that these differences occurred in two blocks, B1 (*p* = 0.0006) and B2 (*p* = 0.00062), respectively.

### Reaction time

**Fig. 3:**
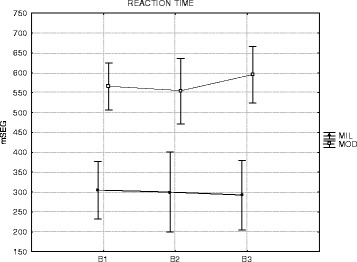
Mean reaction time

Individuals in both groups did not change their reaction times. The conduction of Friedman ANOVA did not yield significant results, *χ* ^2^_(13,2)_ = 0.04347, *p* = 0.97850 (Fig. 3). Nevertheless, the moderate group spent more time to initiate the sequence than the mild group, a difference that was significant for all blocks according to the post hoc Tukey test, B1 (*p* = 0.004), B2 (*p* = 0.017), and B3 (*p* = 0.004).

### Total movement time

**Fig. 4:**
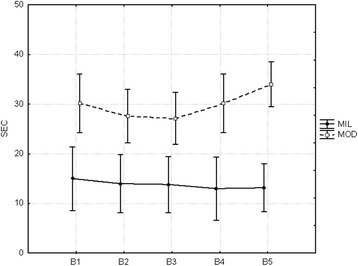
Mean total movement time

The time to complete the task showed slight changes over practice for both groups (Fig. 4). Although the moderate group was slower to complete the task in comparison to the mild group, this result failed to reach statistical significance, *F*_(1,11)_ = 4.4776, *p* = 0.05797. There was a statistical significant increase in the total movement time for the moderate group, from the third block on (B3 to B5; *p* = 0.005).

### Variability of sequence

**Fig. 5:**
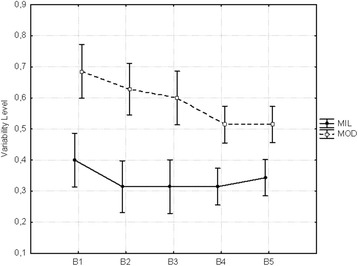
Mean variability of sequence

The variability of the sequence decreased for the moderate group but not for the mild group, F_(1,11)_ = 11.757, *p* = 0.0050 (Fig. 5). There was a discrete difference between the groups with the mild group being more consistent than the moderate group. This result indicates that the mild group showed one to two different sequences in five trials. The moderate group instead showed three to four different sequences in five trials.

### Relative timing variability

**Fig. 6:**
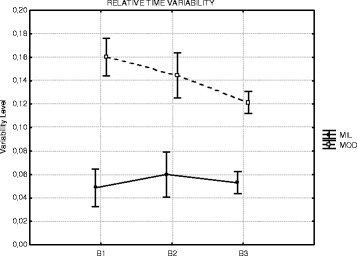
Mean relative timing variability

The time structure of the sequence was more consistent for the mild group than that for the moderate group, though this did not reach statistical significance, *χ* ^2^_(14,2)_ = 1.884, *p* = 0.3897 (Fig. 6).

## Discussion

In the present study, we addressed three questions: (1) Will Down syndrome individuals learn a sequential task? (2) Will they show the formation of an action program to perform a sequential task? (3) Will the formation of an action program be constrained by the level of mental handicap (mild and moderate Down syndrome)? The first question could not be properly answered. The overall performance did not change over practice for both groups that indicate a likely absence of practice effect. Further studies should increase the number of practice trials and also include retention or transfer test to disentangle performance and learning effects. In spite of this, it is worth looking for group differences in regard to what they might concern to the structure of the program.

There were marked performance differences between the mild and moderate groups that suggest that the level of handicap has a motor dimension rather than only an intellectual dimension which is the basis for handicap categorization. When one considers the mean total movement time and the mean overall sequence error, as indicators of measures of speed and accuracy, respectively, it can be argued that the moderate group while showing a gradual increase in accuracy to perform the sequence (decrease of the mean overall sequence error) has also shown an increase in speed (decrease in the mean total movement time) but up to a point, when the response duration starts to increase again. It is as if the individuals with Moderate DS reduce their speed in order to be more accurate in performing the sequence. The mild group did not show this speed-accuracy trade-off. Down syndrome individuals have problems to structure spatially motor sequences also due to limitations associated with memory problems and selective attention (Reid 1980; Horgan 1983; Inui et al. 1995; Lanfranchi et al. 2015). These difficulties would be associated not only with information storage but also with an inability to recognize stimuli and determine strategies for information storage. However, these problems may not be in the same dimension for all DS individuals. There is now a reasonable body of evidence indicating that the difficulties just described may be associated with the level of intellectual deficit (Conners et al. 2008; Frenkel and Bourdin 2010; Edgin et al. 2010).

In regard to the second question, the variability of the sequence did not change over practice for both groups. However, there were differences between their variability that suggests the mild group implemented a more consistent action program. In five possible alternatives, the mild Down syndrome individuals showed one or two different sequences. The moderate group was very variable with three to four sequences within five trials. This might indicate that the moderate DS individuals were not very clear on what sequence to set. In spite of the limited number of trials, the results from the relative timing variability contribute to this interpretation. The mild group showed less variability in the timing of the sequence that is in accord with a more stable representation of the action. In this regard, it is interesting to note that the moderate group although more variable than the mild group did show a decrease in variability with the first two-thirds of practice. The search for a more stable relative timing is hallmark for better performance in serial skills; hence, DS individuals with moderate handicap are not different than their counterparts from the mild level and typically developing individuals.

In previous studies, we found that the variability of the sequence is good predictor of the formation of an action program (Manoel et al. 2002; 2011). DS individuals are indeed more variable than typically developing individuals while practicing a graphic skill (cf. Gimenez et al. 2006). In the present study, we have some evidence (from variability of sequence and relative timing variability) that the mild DS individuals showed a more stable action program in comparison with moderate DS individuals which takes us to the last research question concerned with the role of the level of handicap in the skill acquisition.

There is a limitation on what can be said in regard to acquisition because no statistical significant effects were found in regard to practice. Nevertheless, we did find some significant results to support that DS individuals with different levels of handicap will show different ways to structure their actions. Moderate DS individuals are not only more variable in the sequencing and timing of the sequential aiming movements; they showed also longer reaction times to initiate the action in comparison with the reaction times of the mild group. Considering the one-target advantage rationale, longer reaction times mean that moderate DS individual spent more time to select a plan of the elements of motor programming. Hence, the level of handicap interacts with the planning of the sequence; the moderate DS individuals spent more central processing time to plan and access the sequence of aiming movements. A multitarget task demands more central processing time for DS individuals with moderate levels.

The present results need to be explored in further studies considering the relative temporal organization of the sequence which is a key element in identifying the action program particularly for individuals with Down syndrome (Henderson et al. 1981; Gimenez et al. 2006). One of the assumptions is that, in addition to memory limitations for ordering components, establishing temporal relations between them would be crucial as well (Conners et al. 2008; Edgin et al. 2010; Michael et al. 2012).

Topographic particularities in areas such as the hippocampus, pre-motor cortex, and motor cortex in DS individuals with moderate deficiency levels may be considered determinants for differences in sequencing performance. In particular, regarding the temporal aspects, the specificities associated to the cerebellum may have contributed to the inferiority of the performance in this group (Stoodley and Schmahmann 2009).

The results found in the present study corroborate with previous research that points to the interaction between levels of cognitive development and motor performance in populations with Down syndrome in sequential motor tasks (Schurink et al. 2012; Chen et al. 2014).

The detailed analysis of the temporal relationship between taps can help the understanding of the formation of the action program. This would help to test also other hypothesis according to which the central mechanisms underlying timing problems are also associated with problems in speech identified in many individuals of this population (Bussy et al. 2011).

The present study needs to be replicated increasing the amount of practice with a retention or transfer test to disentangle performance and learning effects. Another aspect to be taken into consideration is whether the problem is one of attention rather than forming a program. Every subject had to pay attention to the numbers indicating which plate should be touched and in what order. Attention here is also associated with short-term memory and spatial orientation as well. The memory was tested in its span; each sequence had five items to be stored with different locations varying sides (left-right) and positions (far-close).

Despite the assumptions associated to the behavior of handicapped people that permeated the present work, it is understood that the use of a control group in future studies composed of typical individuals could contribute to a better understanding of the mechanisms involved in learning the motor task, how the temporal organization among the elements of action.

## Conclusions

The understanding of the behavior of handicapped individuals could benefit from a strategy that attempts to unveil their motor control adaptations rather than looking for differences between them and the typically developing individuals. In the present study, we investigated how DS individuals with two levels of handicap learned a sequential task. The study failed to provide enough practice experience to secure a better chance for them to learn the task. Nevertheless, there is a trend for performance differences between the two DS groups that might suggest different motor control strategies used by each group. Further studies need to consider at least four aspects: (1) increase the amount of practice and include a retention and transfer task to disentangle performance and learning effects; (2) include tasks with different number of items to check for the demand on the span of the short-term memory; (3) include tasks with various spatial configuration, i.e., with and without movement reversals and changes in the movement direction to test for online programming.
